# A Systems Engineering Perspective on Homeostasis and Disease

**DOI:** 10.3389/fbioe.2013.00006

**Published:** 2013-09-09

**Authors:** Yoram Vodovotz, Gary An, Ioannis P. Androulakis

**Affiliations:** ^1^Department of Surgery, University of Pittsburgh, Pittsburgh, PA, USA; ^2^Center for Inflammation and Regenerative Modeling, McGowan Institute for Regenerative Medicine, University of Pittsburgh, Pittsburgh, PA, USA; ^3^Department of Surgery, The University of Chicago, Chicago, IL, USA; ^4^Department of Biomedical Engineering, Rutgers University, Piscataway, NJ, USA; ^5^Department of Chemical and Biochemical Engineering, Rutgers University, Piscataway, NJ, USA; ^6^Department of Surgery, Rutgers Robert Wood Johnson Medical School, New Brunswick, NJ, USA

**Keywords:** systems biology, inflammation, trauma, systems engineering, humans

## Abstract

Engineered systems are coupled networks of interacting sub-systems, whose dynamics are constrained to requirements of robustness and flexibility. They have evolved by design to optimize function in a changing environment and maintain responses within ranges. Analysis, synthesis, and design of complex supply chains aim to identify and explore the laws governing optimally integrated systems. Optimality expresses balance between conflicting objectives while resiliency results from dynamic interactions among elements. Our increasing understanding of life’s multi-scale architecture suggests that living systems share similar characteristics with much to be learned about biological complexity from engineered systems. If health reflects a dynamically stable integration of molecules, cell, tissues, and organs; disease indicates displacement compensated for and corrected by activation and combination of feedback mechanisms through interconnected networks. In this article, we draw analogies between concepts in systems engineering and conceptual models of health and disease; establish connections between these concepts and physiologic modeling; and describe how these mirror onto the physiological counterparts of engineered systems.

## Introduction

Genome sequencing and high-throughput technologies have revolutionized our approach to addressing biological questions. The advent of these methods has created the opportunity to perturb biological systems and observe genome-scale cellular responses (Ideker et al., 2001; Huang et al., 2005). *Systems biology* was introduced as a means by which to describe scientific inquiries through a global approach to elucidate, quantify, model, and potentially reverse engineer biological processes and mechanisms (Cassman et al., 2007; Rigoutsos and Stephanopoulos, 2007). Systems biology has allowed us to address the question of how cells behave as integrated systems rather than as mere sums of their parts (Wiley et al., 2003; Palsson, 2011). Mathematical formalisms have been developed that use mechanistic information and physiological knowledge to simulate behaviors at the organism level and provide a mechanistic basis for pathophysiology (An et al., 2008). This development was, to a great extent, driven by a desire to “… encourage [physicians] to make the subtle but important distinction between [clinical] outcomes and [biological] processes” (Buchman, 2009). If health represents a living organism’s ability to maintain stability in the face of changing internal and external environments, then illness can be defined as the failure to accommodate these changes (Ahn et al., 2006). Systems-based research considers living organisms as networks of dynamic components with identified boundaries and rules that guide their response (An et al., 2008; Foteinou et al., 2009a,b; Vodovotz, 2010; McGuire et al., 2011). Given the high inter-dependence among the constituent parts of a living system and the non-intuitiveness of non-linear biological responses, the living organism may be viewed as a structure sharing the fundamental characteristics of “system of systems”: autonomy, synergism, connectivity, diversity and resilience (Sauser et al., 2010).

With our increasing understanding of life’s multi-scale trans-hierarchical architecture, it has been suggested that living systems share characteristics common to engineered systems and that there is much to be learned about biological complexity from engineered systems (Csete and Doyle, 2002; Doyle and Csete, 2011). This is not to say that biological systems are engineered systems: biological systems are clearly distinct and different by virtue of having resulting from evolution as opposed to design. However, there are some similarities between their consequent organization and that of engineered systems that can provide useful insights (D’Onofrio and An, 2010). For instance, engineered systems can be perceived as coupled networks of interacting sub-systems, whose dynamics are constrained to tight requirements of robustness (to maintain safe operation) on one hand, and maintaining a certain degree of flexibility to accommodate changeover on the other. The aim of analysis, synthesis, and design of complex supply chains is to identify the laws governing optimally integrated systems. Optimality of operations is not a uniquely defined property and usually expresses the decision maker’s balance between alternative, often conflicting, objectives. Both biological and engineered complex constructs have evolved through multiple iterations, the former by natural processes and the latter by design, to optimize function in a dynamically changing environment by maintaining systemic responses within acceptable ranges. Deviation from these limits leads to possibly irreversible damage. Stability and resiliency of these constructs results from dynamic interactions among constitutive elements. The precise definition and prediction of complex outcomes dependent on these traits is critical in the diagnosis and treatment of many disease processes, such as inflammatory diseases (Vodovotz and An, 2013).

In this article, we attempt to draw analogies between fundamental concepts pervasive in systems engineering theory and practice and conceptual/theoretical models of health and disease, with particular examples in the setting of inflammatory diseases. We opt to establish connections between these concepts and physiologic modeling, as well as how these concepts mirror themselves onto critical aspects of notional physiological counterparts of engineered systems.

## A Systems View of Homeostasis

In the 1920s, Kahn presented his rendition of a human and his fundamental physiological functions in the form of interconnected processing units forming an *industriepalast* (a chemical plant) (Debschitz et al., 2009). These units exchange mass and energy, among themselves and with the environment, so as to maintain proper function by appropriate physico-chemical transformations of mass while producing and consuming energy. Around the same time period, Cannon was beginning to lay the foundations, based on the earlier work of Bernard, of the concept of *homeostasis* (Cannon, 1929; Gross, 1998). This is a word of Greek origin: ὅμοιος, *hómoios*, similar, and στάσις, *stásis*, standing still, nowadays defined as the “relatively stable condition of extracellular fluids that results from regulatory systems actions (Windmaier et al., 2004).” Homeostasis posits that living organisms are composed of an intricate web of “alive” parts that exist in and are surrounded by an internal (to the organism) environment. The fixity, or constancy, of this *milieu intérieur* (internal environment, referred to the extra-cellular fluids that provide stability to the organs), and was thought to be necessary for a free and independent life. Cannon postulated that it is the regulation of homeostasis that endows living organisms with the ability to evolve, adapt, and survive. The importance of the homeostasis hypothesis was the realization that “(…) the fixity of the milieu supposes a perfection of the organism such that the external variations are at each instant compensated for and equilibrated…. All of the vital mechanisms however varied they may be, have always one goal, to maintain the uniformity of the conditions of life in the internal environment…. The stability of the internal environment is the condition for the free and independent life.” These fundamental concepts are still being studied and analyzed as we have recently gained the ability to assess globally the nature of the “factors” and “controls” at a cellular and molecular level. The terminology that is now used has evolved from the time of Bernard and Cannon, and we now talk about the concept that the “mechanisms for maintaining this stability [of the *milieu intérieur*] require sensors to recognize discrepancies between the sensed and set of acceptable values and require effectors that reduce those discrepancies – i.e., negative feedback systems (Goldstein and Kopin, 2007).”

We note that, nearly 100 years ago, physiologists were using terms that are quite common in systems engineering parlance such as: open system, disturbance, automatic adjustment, negative feedback, steady state, signal detectors, a processor, and an effector organ controlled through “negative feedback servo-mechanisms” (Buchman, 1996) to imply the existence of control architectures that dissipate disturbances so as to maintain “good health” (homeostasis). By extension, therefore, if good health reflects a dynamically stable and harmonic integration of molecules, cell, tissues, and organs, then disease indicates displacement which is compensated for and corrected by the appropriate activation and combination of feedback mechanisms through interconnected networks (Buchman, 2002).

## Systems Engineering Principles in the Context of Physiological Modeling

In the setting of engineered systems, modeling and simulation complements theory and experimentation since, with advances in computational power, mathematical models enhance the ability of engineers to manage complexity, to explore new solutions efficiently and effectively, and, potentially, to increase the speed of innovation (Quarteroni, 2009). This approach has had, arguably, significant impact in (operational or process) systems engineering, a specific field of engineering that looks beyond individual (sub-)structures and focuses on the design and management of integrated supply chains and cycles composed of intricate networks of interacting components (Braha et al., 2006; Foteinou et al., 2009). Mathematical modeling has been used extensively in physiology in the context of modeling specific functional structures such as organs and organ systems (Keener and Sneyd, 2009). However, the principles and tools associated with the analysis, design, and control of complex engineered systems (complex supply chains) have only recently been utilized to investigate integrated physiological systems at the whole organism level (Butler et al., 2009), as the use of engineering concepts is becoming more familiar to clinicians (Jopling and Buchman, 2012). Engineers have used mathematical models to derive novel processes, to gain efficiency in existing processes, and to analyze trade-offs. In a similar vein, clinicians have begun to appreciate the potential advantages of model-based approaches for describing the true roles of individual components within complex biological systems, raising the possibility of rational manipulation aimed at improve diagnosis and care of patients. The sections that follow aim at discussing how key concepts defining the analysis of engineered supply chains and demonstrate how these are slowly beginning to make their appearance in the context of our efforts to better analyze health and disease.

## Operability and Flexibility

Evolution has selected for living organisms with adaptive mechanisms to maintain constancy of the *milieu intérieur* in the face of a changing external environment (Gerlee et al., 2011): physiological parameters are expected to remain within “reasonable limits.” Exceeding these limits would imply either a response compensating for a deviation from the normal range of values, or a need to activate a compensatory response (Buchman, 1996). If conditioning the *milieu intérieur* within its reasonable limits corresponds to the normal operation, then deviations from that (steady) state would result in, or reveal, a disease. Initial interpretations of this concept were qualitative in nature, since no exact measures of stability were available. However, much as advances in technology and computation have enabled a more accurate spatiotemporal characterization of processes and plants, parallel technological developments have enabled physicians to improve their quantification of “deviation from norm” (Rixen et al., 1995).

The need to handle operational trade-offs often implies a need for feedback control mechanisms that balance appropriate functions. This notional picture, characteristic of control architectures in a plant (Fisher et al., 1988), is equivalent to the mental picture Cannon had created in describing his homeostatic control mechanisms. Flexibility also manifests itself in the ability of the system to activate alternative routes to satisfy changing demands. Flexible manufacturing systems allow engineered supply chains to react to change by switching “machines” or “routes” (sequence of machines). The timely bioenergetics switch from mitochondrial ATP production to glycolysis is an example of the flexibility in the metabolic cellular supply chain in living systems (Zhang et al., 2010). Therefore, “fast and smooth changeover” becomes a crucial property of engineered and natural supply chains for maintenance of homeostasis.

The idea of balancing objectives is a pervasive characteristic of biological systems (Kitano, 2010). In Chandra et al. (2011), it was demonstrated, in a manner that could not have been accomplished without the use of mechanistic mathematical modeling, how glycolytic oscillations express an equivalent to the chemical plant’s interplay between *robustness* and *efficiency*. The response to stress is an excellent example of finding the right balance between detrimental extremes: enough to eliminate the stressor but not too much to damage the host (Laroux, 2004). In the context of adaptive immunity (Segel and Bar-Or, 1999), expressed the need for balancing between the beneficial and detrimental effects of noxious chemicals produced to eliminate an invading pathogen. In a recent study on malaria, the authors suggested that the mosquito vector for the malaria parasite, which uses many of the same immune/inflammatory mechanisms as the mammalian host to control parasite growth and hence transmission, likely balances efficient killing with self-damage in a manner that also results in oscillations in key immune mechanisms (Price et al., 2013). Indeed, the inflammatory response itself is a tradeoff between over-activated response to an initiating stimulus (and attendant self-damage) vs. inadequate response (and consequent failure to clear the offending stimulus, be it an infectious agent or an injury (Vodovotz and An, 2009; Vodovotz et al., 2009). This balancing act often expresses competition among various objectives in terms of resource utilization or potential damage to the engineered system. For biological systems, evolution provides a meta-framework in which these trade-offs balance the relationship between long-term costs and short-term benefits and manifest in the resulting organismal control architecture that is “*not designed by an engineer but shaped by a process of tiny tinkering changes* (i.e., evolution)” (Nesse et al., 2007).

## Abnormal Event Detection and Management in Engineered Systems and in Human Disease

Timely detection, diagnosis, and correction of abnormal deviations from the steady state operation of an engineered plant are all critical tasks, particularly if catastrophic deviations can be foreseen while the plant is still operating normally. The overall process of *Abnormal Event Management* (AEM) has been the focal point of active research in the process engineering community. AEM involves a number of critical steps (Venkatasubramanian et al., 2003a), namely: detection on an abnormal event; Identification of its causal origins; selection of appropriate control decision; and implementation of said decision.

Notionally, two major approaches have been proposed for addressing this classical ill-posed inverse problem: *model-based* methods (quantitative and/or qualitative), which presume the existence of logical links between state, observable, and control variables; and *knowledge-based* methods, which do not presume causal relations, but rather attempt to infer them based on prior historical process data (Venkatasubramanian et al., 2003a,b,c).

While diagnosis of a disease may not typically be described using terms such as *abnormal event* and the corresponding treatment may not be considered an *event management*, the reality is that physicians recognize illness by observing changes, or lack thereof, in patterns of vital signs (Buchman, 2010). However, a problem with bedside monitoring is that observing the current state of the patient is equivalent to recording points in a high-dimensional space attainable from myriad different initial conditions (past history). Furthermore, the critical concerns from the point of view of intervention are (a) at what state will the patient end up in the future; and (b) what kind of interventions would restore the patient’s condition or avoid undesirable excursions. In an interesting analog to AEM, not only is the problem definition equivalent, but also the approaches utilized for monitoring and treatment are similar. Emerging methodologies in the clinical setting involve either an attempt to define, and solve, the inverse problem using *model-based* approaches (Zenker et al., 2007) or an attempt to reconcile the patient’s prior history and identify trends in historical data, i.e., *knowledge-based* (Cohen et al., 2010). The importance of steps (ii) and (iii) above really defines the essence of a physician’s dilemma, which aims at distinguishing processes (causal origin) from outcomes (event manifestation) (Buchman, 2009).

A model-based approach becomes more appealing as our knowledge about a system grows, allowing for the application of mechanistic models of system function. This type of modeling approach raises the possibility of inferring the future state of the system based on its characteristic dynamics by identifying warning signals that are harbingers of a deviation from its normal dynamic steady state. Through modeling, it is possible to assess disease state relative to predicted future dynamics rather than relative to the levels of individual biomarkers. Yet there is still also room for improvements in knowledge-based approaches, particularly as high-dimensional clinical data become available. For instance, even a relatively simple disease severity metric based on high-dimensional gene expression data from peripheral blood leukocytes of trauma patients was able to provide some significant associations with outcomes, even after normalizing for currently used ICU scoring systems (Warren et al., 2009). Thus, early prediction of abrupt transitions, before noticeable changes in the state variables, would be of significant translational interest. In that sense, we have explored the application of stability metrics to studying homeostatic and acute response dynamics, including how stability of the system changes with circadian rhythms. This analysis represents a first step toward identifying how the dynamical components of the inflammatory network can be leveraged to predict forthcoming inflammatory “tipping points” (An et al., 2012a,b; Dick et al., 2012) as well as to understand system stability properties more broadly.

## Decentralized Decision Making

Driven by increased complexity, tighter operational constraints and technological advances, the control structures of modern supply chains, deployed to ensure flexibility, and operability, are becoming more elaborate and are adopting plant-wide distributed characteristics. These developments have presented both opportunities for a better overall management as well as challenges to the existing control theories and structures (Jillson and Erik Ydstie, 2007). At an even higher level, the process community has realized the need for integration beyond the plant, thus introducing the concept of the “*smart plant*” (Christofides et al., 2007). In such smart plants, the process, the plant, and the corporation all participate in establishing operating procedures that properly balance the required trade-offs (Christofides et al., 2007). Therefore, disturbances of any kind in an integrated supply (including supply and demand, machine breakdown, policy decisions etc.) chain are detected and handled in both local and integrated manners. The inherently distributed nature of the way processes are monitored in real time introduced new requirements necessitating handling of asynchronous information flow (Androulakis and Reklaitis, 1999; Xu and Bao, 2011) which, in complex supply chains, implies the lack of a central (master) controller, and by extension the lack of a unique guiding goal (or objective).

In an analogous manner, a living organism deals with threats to its homeostasis via means of a multi-hierarchical system combining centralized and decentralized controllers (Segel, 1997; Lee and Bar-Or, 1999). A number of interesting questions emerge related to how the host senses a local deviation from homeostasis, how the host measures performance toward restoring homeostasis, what objective function is used, how the magnitude of response is controlled, and which of the various effectors are activated, to name a few. When the host is faced with an infection or injury, a complex inflammatory response is initiated with localized cellular and molecular responses aiming at locating the invading pathogen and destroying or at least compartmentalizing it. These pro-inflammatory responses are kept in check through the actions of anti-inflammatory cytokines and hormones (Nathan, 2002), as well as fast-acting, centrally controlled neural-based mechanisms (Tracey, 2002; Dick et al., 2012). Regional neural mechanisms (so-called cholinergic anti-inflammatory pathways) (Tracey, 2007) possibly providing direct local regulation of the inflammatory response. Through the analysis of computational models encompassing multiple different pathways of anti-inflammatory activity, ranging from the local transcriptional response in peripheral blood leukocytes to the more general central hormonal response to inflammation, along with crosstalk among these pathways, the relative contributions of these pathways can be assessed quantitatively and the potential for specific therapeutic interventions can be evaluated (Foteinou et al., 2009c; Dick et al., 2012). The existence of a variety of levels of control has led us to hypothesize that loss of stability across any of these distributed levels leads to failure of containment, and that multiple feedback loops propagate the response peripherally and centrally; thus, host deterioration becomes a diffusible ailment (An et al., 2012a,b; Dick et al., 2012).

## Variability: Friend or Foe?

The concept of *constancy* of the internal environment is not meant to imply invariability, as one may have considered it to be in the context of Kahn’s equivalent chemical plant. Rather, constancy implies specific dynamic characteristics, manifesting themselves in the form of “homeostatic rhythms” rather than a “homeostatic flat line” (Achoff, 1981). The most recognizable rhythms are circadian, which refers to biological processes exhibiting inherent oscillations of approximately 24 h periods. Loss of rhythmicity is a key contributing factor to, as well as manifestation of, disease (Lowry, 2009; Chan et al., 2012). Physiologic and biochemical homeostatic rhythmicity induces a temporal predictability of endogenous controls which is “*presumed to confer acute adaptive advantages that likely extend to modulating systemic illness and solid organ function*” (Lowry, 2009; Mavroudis et al., 2013). This is important in that the dynamic characteristics of the communication channels – that is, the characteristics of the *milieu intérieur*, – play a central role in maintaining overall network stability. Thus, illness, either organ-specific or systemic, might result in loss of signal variability which in turn may further compromise adaptability (Lowry and Calvano, 2008), leading researchers to hypothesize that unnatural patterns (i.e., ones that have lost rhythmicity), such as continuous feeding (Gale et al., 2012), could adversely affect the host. In the broader context of stress (any perceived threat to homeostasis), optimal basal activity of the stress system may be necessary for a sense of well-being, whereas either excessive or inadequate stress activity can lead to pathological conditions (Chrousos, 2009). The variability inherent in physiological systems is often driven by the presence of negative feedback control systems (Novak and Tyson, 2008) which are critical in determining stability and responsiveness (Savageau, 1974). Thus stability, movement among steady states, responsiveness, and robustness are all intertwined.

In a series of theoretical papers, Chauvet (1993a,b,c) argue that the increased association among functional biological groups, through communication via the fluids in the extracellular internal environment, enables increases in the *domain of stability* of a biological system. Expanding further on this idea, it was subsequently hypothesized that disruption of inter-organ communication which results in uncoupling and isolation, ultimately progresses to irreversible damage (Godin and Buchman, 1996). Theoretical analyses demonstrated such long range connectivity could improve recovery (Hubler and Buchman, 2008). It has been hypothesized that changes in the dynamic characteristics of physiological signals are reflective of the internal environment and also of communication among organs, and therefore these dynamic changes either induce host dysfunction or are themselves manifestations of host dysfunction (Godin and Buchman, 1996). Thus, illness, either organ-specific or systemic, might result in loss of signal variability which in turn may further compromise adaptability (Lowry and Calvano, 2008).

If the *industriepalast* is now considered to be an integrated system which operates under the influences of a periodically varying internal environment in order to improve the stability and operability of its homeostasis, it is worth noting that the idea of operating chemical reactors under time varying conditions, in order to explore intrinsic non-linearities, has been argued for a long time. Ray (1968) recognized the implications of periodically varying monomer concentration in producing less dispersed molecular weight distributions for the same average polymer chain length. Renken (1972) discussed theoretical models of increased selectivity in chemical reactors through periodic operation. Bailey (1974) nicely summarized these early theoretical results exploring the hypothesis that (cylic) fluctuations could in fact be considered desirable operating policy and not necessarily disturbances which ought to be eliminated through appropriate control actions. It was later reported (Bailey et al., 1971) that better performance in continuous reactors is the result of operating under fast switching of process variables vs. operating them at the optimum steady state. At the heart of all this early work was the fact that non-linearities induce dynamics that may benefit from the presence of internal dynamics. In the context of modeling components of the inflammatory response, we recently analyzed a coupled model of the HPA axis and the glucocorticoid signaling pathway which, under certain parameter regimes, exhibits ultradian rhythms in glucocorticoid levels propagating through to the pulsatile transcription of glucocorticoid-responsive genes (Scheff et al., 2012). Due to the non-linear binding kinetics involved in glucocorticoid signal transduction, oscillatory HPA axis output allows for the maintenance of low levels of homeostatic responses to glucocorticoids while retaining acute responsiveness to stress, and furthermore the level of peak stress responsiveness correlates with the amplitude of ultradian oscillations. It was hypothesized in Mavroudis et al. (2012) that the circadian rhythmicity in cortisol secretion plays an important role in maintaining synchronicity of clock gene expression, thus establishing robust rhythms in the dynamics of peripheral clock genes. In the context of a surrogate model of systemic inflammation we hypothesized (Scheff et al., 2010; Nguyen et al., 2013) that diurnal rhythms entrained by the cyclic production of the hormones cortisol and melatonin express the interplay between inflammation and circadian rhythms. The mathematical models reproduced diverse sets of experimental data and clinical observations concerning the temporal sensitivity of the inflammatory response (Haimovich et al., 2010). This concept of a positive association between rhythmicity, variability, and performance is exemplified in the context of heart rate variability (HRV), which refers to the quantification of variability in the beat-to-beat functioning of the heart. It was furthermore demonstrated that internal rhythmicity does manifest itself at the systemic level through HRV, while its loss is an indication of stress perturbing the host away from homeostasis (Foteinou et al., 2010; Scheff et al., 2011).

## Concluding Remarks

Both physicians and engineers attempt to propose technical solutions to practical problems (Heller et al., 2008). In a physician’s case, the problems are biological, and manifest at the clinically relevant organismal level (i.e., a person is “sick”). A critical goal of systems-based biological research is to convert novel insights from basic science into clinically relevant actions related to disease prevention and diagnosis, eventually enabling physicians to identify and evaluate treatment strategies (Weston and Hood, 2004; Vodovotz et al., 2008; Foteinou et al., 2009a; Parker and Clermont, 2010; Tian et al., 2012). Integrated initiatives are valuable in uncovering the mechanisms underpinning the progression of human diseases. The advent of high-throughput technologies has enabled the generation of massive amounts of biological data at an unprecedented rate, facilitating a dramatic increase in the degree of quantification applied to modern biological research (Beard et al., 2005). Despite the explosion of such high-dimensional datasets, the complex, non-linear organization and regulation of biological systems too often defy intuitive predictions, and require the development of computational models in order to gain an understanding of the systems’ functions (Vodovotz and Billiar, 2013). Central to this integrative systems-based approach is the identification of the critical components and interactions that give rise to the emergent host response. Such computational models are not, however, intrinsically useful in a clinical context, and therefore they must be structured in manners that allow them to both leverage clinically obtainable data and ultimately produce clinically useful predictions (Vodovotz et al., 2007; Vodovotz, 2010). In this perspective article, we have attempted to establish conceptual links between fundamentals principles in systems engineering science and their potential links to theoretical models of health and disease. We hypothesize that these principles in the context of the analysis, synthesis, design, control, and operation of complex engineered systems (and supply chains) mirror themselves onto critical aspects of their notional physiological counterparts.

## Conflict of Interest Statement

The authors declare that the research was conducted in the absence of any commercial or financial relationships that could be construed as a potential conflict of interest.
